# Supplementary material for: New drug submissions in Canada and a comparison with the Food and Drug Administration and the European Medicines Agency: Cross-sectional analysis
Source: PLoS One. 2023 Jun 15;18(6):e0286802. doi: 10.1371/journal.pone.0286802 (PMC10270583; doi:10.1371/journal.pone.0286802)
Supplement: S1 Table — (DOCX) [file pone.0286802.s003.docx]

**Supplementary Table: Summary of pivotal information considered by Health Canada, FDA and EMA in decision-making**

| **Generic name** | **Health Canada** | **FDA** | **EMA** |
| --- | --- | --- | --- |
| Aducanumab | The pivotal safety and efficacy information consisted of two identically designed phase 3 clinical trials that were completed contemporaneously. | Studies 301 and 302 were identically designed, randomized, double-blind, placebo-controlled studies each comparing two doses (low dose and high dose) of aducanumab to placebo over 18 months in patients at the early stages of symptomatic Alzheimer’s disease. | Study 302 is presented by the Applicant as the pivotal study for efficacy accompanied by supportive Studies 103 and 301. Studies 301 and 302 were identically designed and planned as Phase 3 studies. |
| Alvimopan | The efficacy of alvimopan was derived from six pivotal and supportive clinical efficacy multi-centre, randomized, double-blind, placebo-controlled, parallel group clinical trials in subjects receiving opioid analgesia for postoperative pain management following surgeries…Overall, a total of 2,154 patients underwent a surgery that included a bowel resection (n = 1,877 BR; n = 273 RC). | The phase 3 clinical trials to support the efficacy of alvimopan in the treatment of post- operative ileus in the bowel resection surgery population included the following five POI trials with 1877 patients in the efficacy database [of which 953 (50.8%) and 924 (49.2%) patients received the 12 mg alvimopan dose and placebo, respectively]. |  |
| Amisulpride | This New Drug Submission was submitted through the Submission Relying on Third-Party Data regulatory pathway. The sponsor submitted quality, non-clinical, and clinical data packages. | Efficacy of amisulpride for prevention of postoperative nausea and vomiting, either alone or in combination with an antiemetic of a different class, was demonstrated in two randomized, double-blind, placebo-controlled, multi-center trials in patients undergoing general anesthesia for elective surgery (Study DP10015 and Study DP10017). |  |
| Ataluren* | The pivotal clinical safety and efficacy information consisted of the following: a Phase 2a (Study 004) and a Phase 2b trial (Study 007). |  | A phase 2b efficacy and safety study of PTC124 in subjects with non-sense-mutation-mediated Duchenne and Becker muscular dystrophy. The main clinical study 007 was performed as multicentre, randomised, double-blind and placebo- controlled. |
| Cilostazol | This New Drug Submission was submitted through the Submission Relying on Third-Party Data regulatory pathway. The sponsor submitted quality, clinical and non-clinical data packages. |  |  |
| Cinnarizine, dimenhydrinate | The primary endpoint of all the pivotal trials was the Mean Vertigo Symptom (MVS) score or the Total Vertigo Symptom score. |  |  |
| Emapalumab | The pivotal evidence for safety and efficacy were provided in the form of a phase 2/3, single-arm, open-label trial of Gamifant, administered on a background of dexamethasone, in pediatric patients with pHLH who had received prior treatments. The study enrolled 27 treatment-experienced patients and 7 treatment-naïve patients. | NI-0501-04: A phase 2/3, open-label, single-arm, multicenter study to assess safety, tolerability, pharmacokinetics and efficacy of intravenous multiple administrations of NI-0501 (emapalumab)… Study NI-0501-05 included 28 patients from study NI-0501-04 and seven patients with pHLH who received emapalumab in the compassionate use program. | The pivotal trial to establish the efficacy and safety is trial NI-0501-04. This study…[was] a single arm Phase 2/3 study. Upon completion of the NI-0501-04 study, patients were invited to participate in Study NI-0501-05 for long-term follow-up for 1 year |
| Finerenone | The sponsor submitted quality, clinical and non-clinical data packages, as well as labelling information. |  | This application is based on efficacy data obtained from the…pivotal Phase III study: FIDELIO-DKD (Study 16244). |
| Human heterologous liver cells | The pivotal clinical information consisted of data from 22 patients enrolled in two studies (CCD02 and CCD05). Both of them were phase II studies. |  | Two pivotal clinical studies with comparable design (CCD02 in Germany and CCD05 in US/Canada) were performed to assess the safety and efficacy of HHLivC in children with UCDs. |
| Lasmiditan | The sponsor submitted quality, non-clinical, and clinical data packages, as well as labelling components. | The efficacy of lasmiditan was demonstrated in two adequate and well-controlled clinical studies (Studies 301 and 302). | Efficacy data for this submission were drawn primarily from three randomised, double-blind, placebo-controlled Phase 3 trials: Studies 301/LAHJ, 302/LAHK, and LAIJ. |
| Lorcaserin hydrochloride | The pivotal clinical safety and efficacy information consisted of the following: three Phase III studies, two of which were in obese patients without Type 2 Diabetes (BLOOM and BLOSSOM) and a Phase III trial in adults with type 2 diabetes (BLOOM-DM). | No information on FDA website. |  |
| Panobinostat | The panobinostat clinical development program in multiple myeloma focused on panobinostat in combination with bortezomib and dexamethasone, and includes a Phase III study, one supportive Phase II study, and safety and preliminary efficacy data from the dose expansion phase of a Phase Ib study. The Phase III study was a randomized, double-blind, placebo controlled study. | Efficacy and safety of pabinostat was evaluated in one randomized trial (LBH589D2308, abbreviated as D2308) and two single-arm trials (a Phase 1b dose finding study of pabinostat and bortezomib in patients with multiple myeloma and a Phase 2 single-arm trial of pabinostat and bortezomib and dexamethasone in 55 patients with relapsed and bortezomib-refractory multiple myeloma). | Study CLBH589D2308 was a multicenter, randomized, double-blind, placebo-controlled phase III study of panobinostat in combination with bortezomib and dexamethasone in patients with relapsed multiple myeloma. |
| Roxadustat | The sponsor submitted quality, clinical and non-clinical data packages. |  | Study Nos. FGCL-SM4592-016, FGCL-SM4592-017, FGCL- 4592-041, FGCL-4592-040, FGCL-4592-053, FGCL-4592-047, 1517-CL-0304, FGCL-4592-048 were submitted as the main clinical phase 3 studies in support of this application. Four of them were conducted on non-dialysis dependent (NDD) patients and four were conducted on dialysis dependent (DD) patients. |
| Sirukumab | The sponsor submitted pre-clinical, clinical, quality and labelling components. |  | There were three pivotal phase 3 RCT: ARA3002, ARA3003, and ARA3005. |
| Volanesorsen | The sponsor relied primarily on a single pivotal clinical trial, study CS6…Study CS6 was a 52-week randomized, double-blind, placebo-controlled trial in 66 patients… 33 patients were treated with volanesorsen. |  | A randomized, double-blind, placebo-controlled, phase 3 study of ISIS 304801 administered subcutaneously to patients with familial chylomicronemia syndrome (FCS) |

*****No information about second submission withdrawn by company.
